# Translating Features to Findings: Deep Learning for Melanoma Subtype Prediction

**DOI:** 10.3390/dermatopathology12040042

**Published:** 2025-11-12

**Authors:** Dorra Guermazi, Sarina Khemchandani, Samer Wahood, Cuong Nguyen, Elie Saliba

**Affiliations:** 1 Department of Dermatology, The Warren Alpert Medical School of Brown University, Providence, RI 02903, USA; sarina_khemchandani@brown.edu (S.K.); samer_wahood@brown.edu (S.W.); elie.saliba01@lau.edu.lb (E.S.); 2 Department of Dermatology, Northwestern University Feinberg School of Medicine, Chicago, IL 60611, USA; cuong.nguyen@northwestern.edu; 3 Department of Dermatology, Gilbert and Rose-Marie Chagoury School of Medicine, Lebanese American University, Beirut 13-5053, Lebanon

**Keywords:** melanoma, deep learning, histopathology, melanoma subtypes, artificial intelligence, dermatopathology, convolutional neural networks, image analysis

## Abstract

Melanoma subtyping plays a vital role in histopathological diagnosis, informing prognosis and, in some cases, guiding targeted therapy. However, conventional histologic classification is constrained by inter-rater reliability, morphologic overlap, and the underrepresentation of rare subtypes. Deep learning (DL)—particularly convolutional neural networks (CNNs)—presents a compelling opportunity to enhance diagnostic precision and reproducibility through automated analysis of histopathologic slides. This review examines the clinical importance and diagnostic challenges of melanoma subtyping, outlines core DL methodologies in dermatopathology, and synthesizes current advances in applying DL to subtype classification. Pertinent limitations including dataset imbalance, a lack of interpretability, and domain generalizability are discussed. Additionally, emerging directions such as multimodal integration, synthetic data generation, federated learning, and explainable AI are highlighted as potential solutions. As these technologies mature, DL holds considerable promise in advancing melanoma diagnostics and supporting more personalized, accurate, and equitable patient care.

## 1. Introduction

Melanoma, a malignant tumor of melanocytes, is a clinically and biologically heterogeneous disease [1]. Accurate histopathologic subtyping plays an important role in guiding prognosis and, in some cases, informing treatment decisions. Subtypes such as superficial spreading melanoma (SSM), nodular melanoma (NM), and acral lentiginous melanoma (ALM) differ not only in histologic appearance, but also in patterns of spread, mutation profiles, and patient outcomes. However, traditional diagnostic classification relies heavily on visual interpretation of hematoxylin and eosin (H&E) stained slides, which can be subjective and prone to variabilities between readers [2]. Additionally, the increasing complexity of melanoma presentation, coupled with the scarcity of certain subtypes, further exacerbates diagnostic variability and delays in care.

The advent of artificial intelligence (AI), particularly deep learning (DL), has introduced promising tools to enhance diagnostic precision in pathology. DL, especially convolutional neural networks (CNNs), has demonstrated success in a variety of medical imaging tasks, including cancer detection and classification [3]. In the context of dermatopathology, DL offers the potential to improve consistency, efficiency, and reproducibility in melanoma subtyping by learning from large-scale annotated histologic images. This review focuses specifically on the application of deep learning to histology-based melanoma subtyping. We outline the clinical and pathological challenges that motivate computational solutions, describe the underlying DL methods utilized in this context, examine recent developments in the field, and explore future directions and unresolved challenges.

### Search Strategy and Scope

To identify relevant literature, we conducted a structured search of PubMed, Embase, and IEEE Xplore from January 2015 through January 2025, supplemented by manual review of reference lists from included articles. Keywords included combinations of melanoma, subtype, deep learning, convolutional neural networks, histopathology, whole-slide image, multimodal, and artificial intelligence. We focused on studies that specifically addressed melanoma subtyping or related histology-based classification tasks, as well as methodological papers in computational pathology with direct applicability to subtype prediction.

Studies were selected for inclusion based on novelty of approach, availability of validation beyond single-institution datasets, clinical relevance of endpoints (diagnostic accuracy, prognostic prediction, or subtype-specific outcomes), and contribution to conceptual or technical diversity. We did not aim to comprehensively summarize every proof-of-concept but instead highlighted representative works that illustrate key advances, challenges, and future opportunities in the field.

While DL encompasses a wide variety of architectures, our review emphasizes convolutional neural networks (CNNs) because they remain the dominant framework in histopathology image analysis, with the largest body of evidence supporting their performance in melanoma classification [4,5]. Nonetheless, we also note emerging trends, including vision transformers, attention-based multiple instance learning, and hybrid multimodal models, which are increasingly applied to histology and may offer advantages in capturing long-range dependencies or integrating diverse data streams. These trends are discussed in the relevant sections on limitations and future directions.

## 2. Background: Melanoma Subtypes and Diagnostic Complexities

### 2.1. Clinical and Histologic Diversity of Melanoma Subtypes

Melanoma encompasses a biologically and histologically diverse group of malignancies, each with distinct patterns of growth, anatomical predilections, and clinical behavior. The most prevalent subtype is superficial spreading melanoma (SSM), which typically arises on intermittently sun-exposed skin, such as the trunk and extremities [6]. Histologically, SSM is characterized by a radial growth phase involving the lateral proliferation of atypical melanocytes at the dermoepidermal junction, often accompanied by pagetoid spread [6]. In contrast, nodular melanoma (NM) typically presents as a vertically growing tumor without a preceding radial phase. Clinically, it appears as a rapidly enlarging nodule and is often associated with a more aggressive course and worse prognosis due to earlier invasion into deeper dermal layers [6].

Other subtypes display unique anatomical and epidemiological features. Acral lentiginous melanoma (ALM) is more frequently observed in patients with darker skin types and occurs on glabrous skin—most commonly the palms, soles, and subungual regions [7]. ALM is often diagnosed at a more advanced stage, contributing to worse outcomes [8]. Lentigo maligna melanoma (LMM) arises in the setting of chronic sun exposure, particularly in older adults, and is typically located on the head and neck [9]. It typically progresses slowly and may remain in a prolonged in situ phase; however once invasive, it can exhibit aggressive behavior [9]. Desmoplastic melanoma (DM) represents a rare subtype characterized by dense fibrous stroma and a paucity of melanocytic features [10]. Its histology often mimics scar tissue, neurofibromas, or fibrosing dermatologic conditions, leading to frequent misclassification. The clinical diversity and variable presentation of these subtypes necessitate accurate histologic discrimination for effective prognostication and management. These melanoma subtypes, key features, common sites, and challenges are summarized in Table 1.

### 2.2. Diagnostic Challenges and the Need for Decision Support

Accurate melanoma diagnosis carries significant prognostic and therapeutic implications yet remains one of the most challenging tasks in dermatopathology. A large retrospective cohort study published in 2024 identified an inter-rater diagnostic discordance rate of 23.7% for melanocytic lesions, highlighting persistent challenges in consistent classification [11]. Variability has been studied in the reporting of crucial prognostic features including Breslow thickness, ulceration, and regression [12]. Additionally, histologic ambiguity is frequently observed in routine practice as lesions often do not neatly fit into a single subtype or may evolve over time, further complicating definitive classification [13]. Rare subtypes such as ALM and DM are particularly prone to underdiagnosis or misdiagnosis due to their subtle or atypical features [14].

Moreover, the boundaries between subtypes are not always clearly defined, and criteria can vary among institutions and pathologists. This diagnostic uncertainty is further exacerbated by the limited exposure to rare variants during dermatopathology training, leading to decreased confidence and consistency in real-world diagnosis. Compounding this issue is the fact that many existing histopathology datasets used for research or model training are skewed toward more common subtypes, such as SSM and NM, while underrepresenting rarer forms such as ALM and DM. This lack of balance restricts the generalizability of both human expertise and computational tools.

Given these challenges, there is a need for decision support systems that can aid dermatopathologists in achieving more accurate, reproducible, and subtype-specific diagnoses. Deep learning and other artificial intelligence-based tools have the potential to bridge this gap by identifying diagnostic features, reducing inter-rater reliability, and enhancing diagnostic confidence in complex or borderline cases. These systems may be especially valuable in community or low-resource settings, where access to subspecialty pathology expertise is limited [15].

## 3. Deep Learning Foundations in Dermatopathology

### 3.1. CNNs, Whole-Slide Images, and the Role of Preprocessing

**Figure 1:**
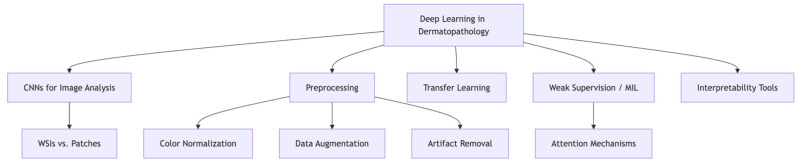
Deep Learning in Dermatopathology.

Deep learning, particularly through convolutional neural networks (CNNs), has become a cornerstone in the field of computational pathology. CNNs are uniquely suited to histologic image analysis due to their ability to extract hierarchical features, ranging from cellular-level morphology to more complex tissue structures. In dermatopathology, CNNs have been utilized for a variety of tasks, including tumor detection and subtype classification, frequently using either whole-slide images (WSIs) or smaller image patches [16]. WSIs offer the advantage of complete tissue context and allow for end-to-end learning across large spatial scales. However, their enormous size poses computational challenges, including memory constraints and long processing times, often necessitating slide tiling strategies and distributed computing. This process is summarized in Figure 1.

In contrast, patch-based approaches reduce computational load and make it feasible to train models on more limited hardware. These patches, typically derived from tiled WSIs, are well suited for capturing localized features such as cellular atypia or mitotic figures. However, they may lose broader contextual information necessary for identifying architectural patterns, which are often critical in histopathological diagnosis. This tradeoff has led to the development of hybrid models that aggregate predictions across multiple patches or integrate features from both global and local views.

Prior to model training, histology images require careful preprocessing to ensure consistency and robustness. One major concern is the variability introduced by differences in staining protocols and imaging hardware. Color normalization techniques, such as the Macenko or Reinhard methods, are commonly employed to align color distributions across images and mitigate staining-related artifacts. Data augmentation strategies, including geometric transformations and color perturbations, expand the effective size of training datasets and help prevent overfitting. Additionally, removing artifacts such as tissue folds, pen marks, or out-of-focus regions is crucial to prevent CNNs from learning spurious patterns unrelated to disease features [17]. These preprocessing steps are foundational in enabling deep learning models to generalize effectively across diverse histologic datasets.

Another critical factor influencing histology image consistency is the variability in how tissue blocks are cut. More experienced histopathology technicians tend to produce thinner, more uniform slides, while less experienced individuals may generate thicker sections. This variability can affect image quality, with thicker sections potentially introducing more artifacts, uneven staining, and optical distortions. Addressing these differences through preprocessing or standardizing cutting protocols helps ensure that deep learning models receive consistent input, thereby enhancing their performance and generalizability.

### 3.2. Transfer Learning, Weak Supervision, and Model Interpretability

Given the limited size of dermatopathology datasets and the high cost of expert annotations, transfer learning has become a standard approach to improving performance. In this paradigm, CNNs are first trained on large, general-purpose image datasets such as ImageNet, where they learn basic visual features including edges, textures, and shapes. These pre-trained weights are then fine-tuned on histologic data, allowing the network to adapt to domain-specific patterns with a smaller amount of labeled data [18]. Architectures such as ResNet, Inception, and EfficientNet are frequently used due to their demonstrated ability to balance depth, accuracy, and computational efficiency.

Another key strategy for overcoming annotation limitations is weak supervision, particularly through multiple instance learning (MIL). In MIL frameworks, models are trained using slide-level labels rather than pixel- or region-level annotations. Each slide is treated as a collection—or “bag”—of image patches, and the model learns to associate certain instances within the bag with the global label [19]. This approach enables large-scale training without the need for exhaustive labeling by expert dermatopathologists.

Interpretability remains a crucial concern in the medical applications of AI [20]. As models are increasingly considered for clinical deployment, understanding how and why a CNN makes a particular prediction is essential. Tools such as Grad-CAM (Gradient-weighted Class Activation Mapping) help visualize the areas of an image that most influenced the model’s decision, offering a bridge between black-box outputs and human understanding [21]. These visualization tools not only support clinical validation but also help identify model failures, biases, or unexpected behaviors—an essential step in establishing trust among dermatopathologists and clinicians.

## 4. Applications of Deep Learning to Melanoma Subtyping

### 4.1. Overview of Approaches and Input Modalities

As mentioned, deep learning has emerged as a promising tool for classifying melanoma subtypes using histopathologic slides. However, existing studies vary widely in terms of dataset scale, labeling strategy, and model design. Most studies have focused on hematoxylin and eosin (H&E)-stained slides, which remain the gold standard for routine histopathological evaluation [22]. Input images typically include either manually selected regions of interest (ROIs) or image tiles derived from whole-slide images (WSIs). In many cases, annotations are weakly supervised, meaning slide-level diagnostic labels are applied without precise cellular or architectural annotation, allowing for scalability but increasing label noise. This inherent label noise can propagate through convolutional neural networks (CNNs) or other deep learning architectures, potentially affecting feature extraction fidelity and compromising model generalization. Consequently, techniques such as multiple instance learning (MIL) and robust loss functions are often employed to mitigate the effects of noisy labels and enhance model performance on histopathological data.

Given the computational demands of WSIs, many models still rely on patch-based classification, wherein small tiles are extracted and independently analyzed, with results later aggregated to produce a slide-level interpretation [23]. However, advances in GPU capabilities and memory efficiency have made it feasible for some groups to develop end-to-end models that process entire WSIs, retaining more contextual information and potentially improving diagnostic accuracy. These models often incorporate attention mechanisms, multi-scale feature extraction, or hierarchical modeling to simulate the decision-making process of a pathologist. Attention mechanisms allow the model to focus on the most relevant parts of the data, enhancing its ability to identify key patterns. Multi-scale feature extraction enables the analysis of information at various resolutions, capturing both fine details and broader contextual features. Hierarchical modeling mirrors the structured approach of pathologists by processing information in layers, progressively refining interpretations to reach accurate conclusions. Together, these techniques aim to replicate the depth and nuance of human diagnostic reasoning.

### 4.2. Subtype-Specific Performance and Limitations

The performance of deep learning models varies markedly across melanoma subtypes. Models generally achieve high accuracy in identifying nodular melanoma (NM) and superficial spreading melanoma (SSM) [24]. These subtypes are characterized by relatively well-defined histologic features—such as expansive dermal growth in NM or pagetoid spread in SSM—and are often well-represented in training datasets, contributing to more reliable classification. In contrast, acral lentiginous melanoma (ALM), lentigo maligna melanoma (LMM), and desmoplastic melanoma (DM) present more formidable challenges. These subtypes often diverge from the typical histologic patterns, manifest in distinct anatomic locations (such as palms, soles, or sun-damaged skin), and may closely resemble benign entities such as nevi, solar lentigines, or scar tissue.

These atypical or subtle features increase both false positive and false negative rates, especially when training data is sparse or unbalanced. In many studies, rarer subtypes are either excluded entirely or grouped under an undifferentiated “other” category, which blunts the ability of models to learn subtype-specific features. This limits not only diagnostic precision but also the clinical utility of the model, as some of the most diagnostically challenging and clinically significant melanomas remain underrepresented or misclassified. These challenges are summarized in Table 2.

### 4.3. Generalizability and Technical Barriers

A major concern in deep learning for melanoma subtyping is model generalizability. When models trained on single-institution datasets are applied to external cohorts, performance often drops significantly due to domain shift—the result of variations in slide staining protocols, scanning equipment, or tissue handling procedures [27]. These shifts can alter pixel-level image properties in ways that are imperceptible to pathologists but disruptive to neural networks. To mitigate these effects, researchers have applied data augmentation, color normalization, and domain adaptation strategies to diversify training distributions and make models more robust to unseen inputs.

Frameworks such as Slideflow, an open-source deep learning pipeline designed for histopathology, have facilitated experimentation with model reproducibility and transparency [28]. Built on platforms such as PyTorch *(1.9.0)* and TensorFlow *(2.5.0)*, these tools support patch extraction, MIL-based training, visualization, and validation across datasets [29]. Notably, progress is being made in improving generalizability through carefully designed multi-institutional studies. For example, a deep learning model trained on hematoxylin and eosin (H&E) slides from 108 melanoma patients across four institutions demonstrated strong predictive performance for disease-specific survival. It was externally validated on an independent cohort of 104 patients from Yale School of Medicine and further tested on 51 patients from Geisinger Health Systems [25]. The model achieved an AUC of 0.905 in the Yale cohort and 0.880 in the Geisinger cohort, with significant Kaplan–Meier survival prediction in the external test set (*p* < 0.0001) [25]. Similarly, Comes et al. (2022) developed a deep learning framework trained on whole slide images of cutaneous melanoma to predict one-year disease-free survival, demonstrating that morphological and spatial features extracted from H&E slides can serve as robust prognostic biomarkers across cohorts [30]. A deep learning model demonstrated its ability to accurately predict disease-specific recurrence (DSS) in melanoma patients based on Kaplan–Meier analysis. The model identified several features, including the density, distribution, and morphology of tumor nuclei, as predictive of survival [26]. Features associated with survival included the density and distribution of lymphocytes as well as tumor nuclear morphology, underscoring the biological relevance of deep-learned representations.

However, despite technical progress, most models remain in the proof-of-concept stage. Few have been externally validated in prospective trials, tested on multi-institutional data, or incorporated into real-time clinical workflows. Moreover, the integration of deep learning tools into pathology practice will require regulatory clearance, interoperability with existing diagnostic platforms, and clear pathways for clinical accountability.

## 5. Limitations and Challenges

### 5.1. Dataset Imbalance and Lack of Representation

One of the most significant limitations in applying deep learning to melanoma subtyping is the pervasive issue of dataset imbalance. Subtypes such as acral lentiginous melanoma (ALM), lentigo maligna melanoma (LMM), and desmoplastic melanoma (DM) are relatively rare compared to nodular and superficial spreading melanoma, and they are often underrepresented in training datasets [31]. As a result, deep learning models trained on imbalanced data struggle to recognize the unique histologic features of these rarer variants. This not only reduces overall diagnostic accuracy but also disproportionately affects the subtypes most likely to be misdiagnosed in clinical practice. Compounding this problem is the fact that many publicly available dermatopathology datasets lack sufficient ethnic and skin type diversity. When models are developed predominantly using slides from lighter skin tones, their generalizability to underrepresented populations becomes questionable, potentially perpetuating healthcare disparities [32]. Addressing these issues will require deliberate efforts to curate more inclusive datasets, apply targeted data augmentation, and explore novel methods such as synthetic data generation using generative adversarial networks (GANs) or diffusion models to simulate rare subtype appearances. GANs work by training two neural networks in tandem—a generator that creates synthetic data and a discriminator that evaluates its authenticity—while diffusion models generate data by iteratively refining random noise until it closely resembles real-world examples.

Addressing the underrepresentation of acral lentiginous melanoma, lentigo maligna melanoma, and desmoplastic melanoma requires deliberate methodological strategies. Class-balanced and focal loss functions can mitigate model bias toward majority subtypes, while reweighting schemes adjust training to better reflect the clinical importance of rare categories. Few-shot and zero-shot learning techniques provide a pathway for recognizing underrepresented subtypes with only a limited number of annotated examples. Preliminary evidence suggests that these strategies may be particularly beneficial for acral and desmo-plastic lesions, where data scarcity is most pronounced. Synthetic augmentation, utilizing generative adversarial networks (GANs) and diffusion models, can enhance the apparent sample size of rare classes. However, the fidelity of these images must be rigorously evaluated [33]. Quantitative metrics such as Fréchet Inception Distance (FID) and precision–recall scores can benchmark image realism, while qualitative “Turing tests” involving blinded dermatopathologists provide additional safeguards against subtle artifacts. Stress testing models against deliberately perturbed or spurious features can further ensure that performance gains are biologically meaningful rather than driven by confounders [34]. Together, these strategies represent a pragmatic roadmap for improving rare subtype recognition while minimizing the risks associated with synthetic data.

### 5.2. Model Explainability and Clinical Trust

Another major challenge lies in the explainability of deep learning models. Despite their strong performance in many classification tasks, CNNs are often viewed as “black boxes” because their internal decision-making processes are not transparent to end users [35].

Preliminary work has shown that AI can identify multiple relevant elements on histopathology slides. For instance, a recent multi-task pipeline at Memorial Sloan Kettering utilized convolutional neural networks (CNNs) to simultaneously identify invasive tumor foci, distinguish in situ areas, delineate tissue layers, detect blood vessels and lymph nodes, and even classify mitotic figures as typical or atypical [36]. This shows that a single platform can be trained to detect diverse histologic features. In principle, similar models could be trained on melanoma slides to output all the key synoptic features.

In dermatopathology, where diagnostic decisions carry significant clinical consequences, this lack of interpretability is a substantial barrier to clinical trust and adoption. Tools such as class activation maps (CAMs), Grad-CAM, and other saliency-based visualizations, referring to methods that focus on the most attention-grabbing or prominent features, have been developed to highlight the regions of an image that most influenced a model’s prediction. While these tools can provide insight into model reasoning, they are still evolving and may not align perfectly with the features a pathologist would consider diagnostically relevant [37]. Moreover, the output of these methods can be difficult to interpret or may vary based on small perturbations in input, limiting their reliability in high-stakes environments. For deep learning models to be accepted in routine diagnostic workflows, they must not only demonstrate high accuracy but also offer clear, interpretable, and reproducible explanations for their decisions.

### 5.3. Technical and Operational Barriers to Clinical Integration

From a technical perspective, one of the most persistent barriers to reliable model performance is domain shift. Models trained on slides from a single institution often perform poorly when applied to external datasets, due to differences in slide preparation, staining protocols, scanning resolution, and image compression. These subtle yet impactful variations can significantly degrade performance in unseen settings. Although preprocessing techniques such as color normalization and stain deconvolution can partially mitigate domain shift, no universally accepted solution exists. Cross-institutional validation, multi-site datasets, and domain adaptation algorithms are therefore critical areas of ongoing research [38].

Beyond technical constraints, integrating deep learning into clinical workflows presents several operational and regulatory challenges. Deploying AI tools in healthcare requires extensive external validation, regulatory approval, and careful alignment with existing diagnostic procedures [39]. The question of clinical responsibility also arises—specifically, how to adjudicate decisions when human and machine disagree. Furthermore, ethical and legal concerns must be addressed. These include risks related to patient data privacy, the potential propagation of embedded biases, and the consequences of incorrect or overconfident model predictions, particularly when decisions are made without adequate human oversight [32]. Misclassification of melanoma subtypes could have serious implications for staging, treatment decisions, and prognosis, emphasizing the need for built-in safeguards, transparency, and human-in-the-loop systems to ensure safe deployment.

## 6. Future Directions and Research Opportunities

### 6.1. Multimodal Modeling and Personalized Predictions

A central opportunity for advancing melanoma subtyping lies in the development of multimodal deep learning models that integrate histologic images with other patient-specific data streams. These may include genomic data (e.g., BRAF or NRAS mutation status), clinical metadata (such as age, lesion location, or immunotherapy history), and non-invasive imaging modalities such as dermoscopy or confocal microscopy. By fusing complementary information, such hybrid models could offer more robust and personalized diagnostic outputs, potentially distinguishing between subtypes that appear histologically similar but differ at the molecular or clinical level. Such integration not only improves classification accuracy but also paves the way for precision dermatopathology, where risk stratification and treatment decisions are tailored to individual profiles.

The latest WHO Classification of Skin Tumors (5th edition) presents a nine-pathway framework for melanoma, which integrates clinical, histopathologic, epidemiological, and molecular dimensions [40]. This schema acknowledges that melanoma subtypes are not solely defined by their morphology, but also by recurrent genetic alterations and their clinical manifestations. For example, BRAF and NRAS mutations are prevalent in superficial spreading and nodular melanomas, NF1 loss is associated with chronic sun-damaged melanomas, KIT mutations are more common in acral and mucosal disease, and GNAQ/GNA11 mutations characterize uveal melanoma. In contrast, our review primarily focuses on the five traditional histopathologic subtypes (SSM, NM, ALM, LMM, DM), which remain the diagnostic benchmarks in routine pathology practice but do not fully encapsulate the molecular heterogeneity recognized in the current WHO schema.

Multimodal deep learning models offer a “natural bridge” between these frameworks. By combining histologic image analysis with genomic, clinical, and epidemiological data, AI systems could classify melanomas in a way that is both morphologically grounded and biologically informed [41]. Such integration would allow models to discern when tumors that appear histologically similar exhibit molecular divergence, aligning computational outputs with the WHO’s nine-pathway classification. Incorporating molecular signatures into these models allows for enhanced prognostic accuracy, facilitation of precision stratification for targeted therapies, and improved histology-based workflows with the evolving genomic taxonomy of melanoma [42].

Concrete examples demonstrate how multimodal fusion can align histology-based AI with the WHO’s nine-pathway schema. For instance, convolutional neural networks can be applied to hematoxylin and eosin slides and then paired with mutational status information like BRAF V600E or NRAS Q61 to distinguish between biologically distinct subsets of superficial spreading and nodular melanoma [43]. Similarly, integrating clinical metadata (patient age, sex, lesion site, and history of immunotherapy) with histology has been shown to improve survival prediction and risk stratification [44]. Beyond clinical and genomic data, complementary imaging modalities such as dermoscopy and reflectance confocal microscopy can provide surface-level and in vivo morphologic cues that enrich purely histologic models.

From a modeling standpoint, multimodal fusion can be implemented in various architectures. Early fusion involves concatenating raw or low-level feature embeddings from different modalities before training a shared classifier. In contrast, late fusion combines modality-specific predictions at the decision level, often using ensemble or weighted averaging techniques [45]. More advanced frameworks employ cross-attention mechanisms to dynamically weight features from histology, genomics, and clinical data, thereby capturing interactions across modalities that may be critical for differentiating histologically similar but genetically divergent melanomas [46]. Public benchmarks for multimodal melanoma analysis remain limited, but resources such as The Cancer Genome Atlas (TCGA-SKCM) and International Skin Imaging Collaboration (ISIC) provide paired histology, genomic, and dermoscopic data streams that can serve as proof-of-concept testbeds [47,48]. Expanding these resources to include detailed clinical metadata and rare subtypes (e.g., acral, desmoplastic) will be essential for evaluating multimodal fusion strategies at scale.

In tandem, synthetic data generation techniques, including generative adversarial networks (GANs) and diffusion models, are emerging as valuable tools for addressing class imbalance. These models can produce realistic, high-resolution synthetic images of underrepresented melanoma subtypes, augmenting training datasets without requiring costly manual annotation. Additionally, few-shot and zero-shot learning techniques may enable models to recognize rare subtypes using only a handful of labeled examples, accelerating progress in low-data settings [49]. This is summarized in Table 3 below.

### 6.2. Federated Learning and Data Privacy

Data access and privacy remain formidable barriers in histologic applications of AI, especially in dermatopathology, where labeled datasets are often small, institution-specific, and governed by strict privacy regulations. Federated learning offers a transformative solution by enabling model training across multiple institutions without transferring raw patient data. Instead, models are trained locally and updated centrally through aggregated weight sharing [50]. This approach preserves patient confidentiality while allowing models to learn from diverse, geographically and demographically varied datasets, thereby improving generalizability and fairness.

Implementing federated learning in dermatopathology could be especially impactful given the heterogeneity of melanoma presentation across populations and institutions [51]. Moreover, federated strategies can be combined with differential privacy techniques to provide formal guarantees against data leakage, strengthening both patient trust and institutional willingness to collaborate.

Beyond data decentralization, emerging privacy-preserving schemes deserve closer examination. Differential privacy introduces controlled noise into model updates to provide formal guarantees against patient re-identification. Secure aggregation ensures that parameter updates from participating sites are encrypted and only revealed in aggregate form [52]. These protections enhance patient confidentiality but are not without tradeoffs. Differential privacy can result in a noticeable decrease in accuracy if noise levels are not precisely adjusted, especially in small or imbalanced melanoma subtypes. Secure aggregation, on the other hand, increases communication expenses and may slow down training due to the need for repeated rounds of encryption and decryption. More broadly, federated learning frameworks can face slower convergence relative to centralized training, and heterogeneous institutional data distributions may exacerbate this problem by reducing gradient alignment across sites [53]. Despite these constraints, privacy-preserving schemes remain critical for enabling collaborative, multi-institutional training in dermatopathology. Anticipating and quantifying these tradeoffs will be essential for realistic evaluation and for designing federated pipelines that balance patient privacy with clinically meaningful performance (Table 3).

### 6.3. Explainable AI and Clinician Confidence

As deep learning tools move closer to clinical application, the need for explainable AI (XAI) becomes more urgent. Beyond heatmaps and saliency overlays, emerging approaches such as concept bottleneck models, attention-based networks, and counterfactual explanations offer more intuitive and structured forms of interpretability. Concept bottlenecks, for example, force models to first predict a set of human-interpretable histologic features—such as pagetoid spread or dermal fibrosis—before arriving at a diagnostic decision. This provides a transparent reasoning path that can be verified or contested by pathologists.

Counterfactual explanations allow users to ask, “What would the model’s output be if this one histologic feature were different?”—a powerful tool for evaluating model sensitivity to specific morphologic cues. Attention mechanisms, meanwhile, help identify which image regions or features contribute most to model confidence, adding an extra layer of interpretability. Together, these techniques could foster greater clinician trust, support quality control, and help bridge the gap between algorithmic decision-making and expert pathology reasoning (Table 3).

### 6.4. Clinical Validation and Standardization

Despite technical advances, clinical translation remains a critical bottleneck. Few models have undergone rigorous prospective evaluation in real-world settings, and even fewer have demonstrated consistent improvements in clinical outcomes. Moving forward, prospective clinical trials will be essential to establish whether deep learning models improve diagnostic accuracy, reduce time to diagnosis, or decrease inter-rater reliability among dermatopathologists. Demonstrating clinical utility is a prerequisite for regulatory approval, payor support, and eventual integration into electronic health records and diagnostic workflows.

Parallel to validation efforts, there is a growing need for standardization and transparency within the histologic AI community. The lack of consistent benchmarking datasets, variable reporting practices, and inconsistent evaluation metrics hampers progress and reproducibility. Open-access initiatives such as the Cancer Genome Atlas (TCGA) and the International Skin Imaging Collaboration (ISIC) serve as models for building shared infrastructure in medical imaging applications of AI. Future collaborative frameworks should prioritize the development of diverse and inclusive training datasets, clearly defined diagnostic endpoints, and transparent model documentation to ensure that tools are not only performant, but also equitable, reliable, and deployable at scale.

To ensure generalizable and equitable performance, melanoma-subtyping studies should adopt standardized lesion descriptors and consistently report the composition of their cohorts across demographic and anatomic strata. At a minimum, datasets should document Fitzpatrick skin type distribution (I–VI), lesion site with explicit designation of acral versus non-acral location (and, when available, mucosal or uveal categories), patient age and gender/sex, as well as tumor characteristics such as stage, Breslow thickness, and ulceration [54]. Technical variables relevant to domain shift, including scanner type, staining protocol, and color normalization method, should also be explicitly reported, since variations in tissue processing and imaging are known to influence deep learning model performance [55]. Given established risks of dataset bias in medical AI studies should pre-specify stratified analyses and incorporate fairness-aware reporting frameworks.

Stratified performance reporting should include, at minimum, measures of discrimination and calibration across Fitzpatrick skin type groupings (I–II, III–IV, V–VI), anatomic categories such as acral versus non-acral melanomas, and patient age (for example, <40 years, 40–64 years, and ≥65 years) and gender/sex [56]. Reporting by institution, scanner, or staining protocol is crucial for quantifying domain shift and should ideally be complemented by site-held-out external validation. Sub-type-level analyses are particularly important for rarer entities like acral lentiginous melanoma, lentigo maligna melanoma, and desmoplastic melanoma, which are often lumped into the “other” category. Performance for these subtypes should be explicitly reported whenever sample sizes permit [57].

From a methodological standpoint, investigators should pre-register analysis plans that outline primary and secondary endpoints, as well as subgroup analyses, in advance. Additionally, they should share code or configuration files through reproducible pipelines to facilitate external verification [58]. Approaches such as reweighting or resampling may help mitigate class imbalance, while few-shot augmentation or synthetic image generation can be considered for extremely rare categories, provided strict human quality assurance is in place. Calibration curves and decision-curve analyses should also be stratified by Fitzpatrick type, lesion site, and demographic subgroups to clarify clinical impact [59]. Finally, when multimodal models incorporate molecular data, subgroup reporting should evaluate whether molecular features reduce observed disparities across Fitzpatrick skin types or acral locations, thereby aligning with the WHO nine-pathway schema and supporting biologically informed classification [60]. Collectively, these practices will operationalize transparent and fairness-aware evaluation, mitigate hidden domain and label shifts, and promote reproducible external validation across diverse populations and care settings.

In addition to standardized reporting and fairness-aware evaluation, studies should explicitly define the intended clinical use case of deep learning systems. Potential roles include triaging routine slides to prioritize suspicious lesions, functioning as a second reviewer to reduce oversight errors, providing quality assurance (QA) checks in community or high-volume settings, or enabling targeted enrichment of rare or diagnostically ambiguous cases such as acral or desmoplastic melanoma [61]. For each use case, appropriate impact metrics should be reported. These extend beyond traditional classification accuracy to include processing and delivery time per case, turnaround time compared with standard practice, and reduction in inter-reviewer variability, which remains a major source of diagnostic discordance [62]. Explicitly linking performance outcomes to these practical endpoints will clarify whether AI tools improve efficiency, consistency, and equity in dermatopathology, and will provide the evidence base necessary for regulatory approval and clinical adoption.

To contextualize the clinical benefit of a model, performance reporting should go beyond accuracy and AUROC. Calibration analyses, such as the Brier score and expected calibration error, quantify how well predicted probabilities align with actual outcome likelihoods. This ensures that risk estimates are reliable for clinical decision-making. Decision-curve analysis should also be incorporated to assess the overall clinical benefit of model use across a range of threshold probabilities [63]. Unlike AUROC, which summarizes discrimination alone, decision-curve analysis identifies the probability thresholds at which an algorithm meaningfully alters clinical management, for example, by reducing unnecessary biopsies or supporting earlier diagnosis [64]. These methods are particularly relevant for melanoma, where the cost of both false positives and false negatives is high, and they provide a more realistic measure of clinical utility in comparison to standard metrics [65]. This is summarized in Table 3 below.

## 7. Discussion

The application of deep learning to melanoma subtyping represents an exciting frontier in computational pathology. Over the past decade, significant strides have been made in using convolutional neural networks to analyze histologic slides and differentiate melanoma subtypes, particularly superficial spreading and nodular melanomas. These advances offer the potential to augment pathologist workflows by improving diagnostic accuracy, reducing variability, and streamlining case triage. However, despite technical progress, the field has not yet achieved the maturity needed for clinical translation. Limitations in dataset diversity, challenges in model interpretability, and a lack of prospective validation continue to hinder widespread adoption.

Importantly, the gaps in melanoma subtyping mirror those encountered in other cancer types. In breast and lung cancer, for example, deep learning models have demonstrated higher degrees of clinical readiness, benefiting from large, standardized datasets, clearer subtype definitions, and well-established molecular correlates. In melanoma, the overlap between histologic subtypes, scarcity of certain presentations, and complex tumor–host interactions make classification more nuanced. The experience from other domains suggests that interdisciplinary collaboration—among dermatopathologists, computational scientists, engineers, and clinicians—will be essential to refine models, design clinically meaningful endpoints, and facilitate responsible deployment.

Emerging tools like polygenic risk scores (PRS) complement histologic AI models. For example, a Dutch cohort study found that familial melanoma cases with higher PRS were more likely to develop multiple primary lesions [66]. Integrating PRS with deep learning-driven histopathologic analysis could lead to more comprehensive risk stratification models that consider genetic susceptibility, subtype morphology, and clinical outcome.

Beyond technical advancements, meaningful progress will hinge on institutional support for digital infrastructure, ethical data sharing, and robust evaluation frameworks. Transparent benchmarking, fair reporting standards, and community-wide datasets are crucial to ensure models are reproducible, generalizable, and equitable. As the field evolves, the pathologist’s role will also need to adapt—from a passive recipient of AI outputs to an expert interpreter, integrator, and steward of these technologies within patient care.

Another crucial aspect of deployment is the implementation of human-in-the-loop checkpoints. In reality, this implies that whenever algorithmic predictions and pathologist impressions diverge, the case should automatically trigger a targeted review instead of relying solely on either source of judgment. Discordance analyses can be reported to identify which melanoma subtypes and clinical contexts are most prone to disagreement—for example, desmoplastic melanoma mimicking scar tissue, acral lesions with subtle lentiginous growth, or lentigo maligna melanoma on sun-damaged skin [67]. In such cases, corrective workflows may include multidisciplinary case conferences, ancillary histochemical or immunohistochemical staining, or reflex molecular testing to resolve the uncertainty [68].

Integration with dermoscopy and clinical photography further enhances the safety and interpretability of human-AI collaboration. For example, acral location, evidence of chronic sun damage, or patient-specific clinical cues can be dynamically used to modify algorithmic reliability thresholds [69]. A lesion from an acral site or an elderly patient with photodamaged skin could prompt the system to reduce its decision confidence, automatically suggesting human review or additional diagnostic steps [70]. These feedback loops ensure that computational models function as augmentative tools rather than replacements, embedding AI within the broader clinico-pathological reasoning process and helping align decision-making with both biological heterogeneity and real-world practice.

In real-world deployment, a crucial consideration is the need for clinico-pathological correlation. Currently, pathologists seamlessly integrate histopathology with various information, including anatomical location, dermoscopic appearance, patient age and gender, medical history, and lesion evolution, to arrive at a definitive diagnosis [71]. Algorithms that produce predictions without reference to this context risk misclassification, particularly for lesions in acral locations or in older patients with extensive sun damage, where histology alone can be ambiguous. Embedding such contextual cues directly into multimodal deep learning models—for example, linking H&E slide features with dermoscopic patterns, lesion site, and BRAF/NRAS mutation status—could approximate the reasoning process of expert dermatopathologists [72]. Alternatively, requiring structured correlation between model output and clinical variables as part of reporting standards may ensure that AI systems are not used in isolation [73]. In both cases, prioritizing the clinico-pathological correlation will be crucial for safe adoption. This approach helps reduce the risk of over-reliance on algorithmic outputs and ensures that computational predictions align with the integrative workflows of modern melanoma diagnosis.

## 8. Conclusions

Deep learning has emerged as a powerful tool in the quest to improve histopathologic subtyping of melanoma. While current models demonstrate promising accuracy in classifying more prevalent subtypes, limitations in dataset diversity, model interpretability, and real-world generalizability prevent their routine clinical use. Bridging the gap between experimental results and clinical integration will require not only technical innovation but also rigorous validation, standardization, and interdisciplinary collaboration.

Despite these hurdles, the future of AI in dermatopathology is bright. As computational models become more transparent, multimodal, and inclusive of diverse populations, they may serve as valuable adjuncts in melanoma diagnosis—particularly in challenging or ambiguous cases. With continued investment in research, infrastructure, and regulatory frameworks, deep learning holds the potential to enhance the precision, consistency, and equity of melanoma care.

## Figures and Tables

**Table 1:** Melanoma subtypes key features, common sites, and challenges.

Subtype	Key Features	Common Sites	Challenges
SSM	Radial growth, pagetoid spread	Trunk, limbs	Common, but overlaps possible
NM	Vertical growth, rapid progression	Anywhere	Aggressive, sometimes missed early
ALM	Acral sites, lentiginous growth	Palms, soles, nail beds	Often diagnosed late; rare
LMM	Chronic sun exposure, slow-growing	Face, scalp	Long in situ phase; can become invasive
DM	Fibrous stroma, bland histology	Sun-exposed or scar-like areas	Mimics scarring; difficult to identify

**Table 2:** Representative deep-learning studies relevant to melanoma subtyping (or enabling subtype-aware models).

Study (Year)	Subtypes Covered/Task	Dataset	N (Patients/WSIs)	External Validation	Key Limitations
Requa et al., 2023, J Pathol Inform [16]	Detection of 5 lesion types with subtyping and localization (melanocytic lesions are subtyped as benign/mildly/severely atypical; BCC and ASL also subtyped).	Single organization (PathologyWatch); multiple PW labs; private	Supervised subset: 2188 WSIs; Weakly supervised subset: 5161 WSIs; Validation: 250 WSIs (+50 mimickers); Testing: 3821 WSIs (daily cases).	No true external institutional cohort; testing was non-curated PW daily cases.	Industry dataset; internal test only; subtype targets differ from WHO melanoma subtypes (focus on severity/phenotype classes); label noise risk in SSL.
Su et al., 2023, Comp Biol Med (Attention2Minority/SiiMIL) [19]	MIL method targeting small/rare lesion instances—relevant to under-represented melanoma subtypes (e.g., ALM/DM) in principle.	Method paper; datasets vary; largely private.	NR	Usually no melanoma-specific subtype benchmark; focuses on algorithmic advance; needs melanoma-task validation.	
Raza et al., 2024, CMIG (Dual-attention + RL for WSI) [23]	Method for slide- and tile-level attention; applicable to histology classification (not melanoma-specific).	Varies by experiment; typically private; indication toward IHC WSIs in examples.	NR	Often no external medical-domain validation reported; not melanoma-specific; demonstrates technique rather than a melanoma study.	
Kulkarni et al., 2020, Clin Cancer Res [25]	Prognostic modeling from H&E (risk of visceral recurrence/death). Not a subtype classifier per se but informs subtype-aware pipelines.	Multi-institution (4 sites); private	Train: 108 pts (4 institutions). External val: Yale 104 pts. Independent test: Geisinger 51 pts.	Yes—Yale (AUC 0.905) and Geisinger (AUC 0.880); KM *p* < 0.0001.	Prognosis (not subtype labels); slide-level labels; no paired genomics; generalizability beyond studied centers not shown.
Phillips et al., 2019, CVPR Workshops (ISIC) [26]	Segmentation of prognostic tissue structures (epidermis/dermis/tumor) in cutaneous melanoma to enable Breslow measurement (enabling feature for subtype-aware/clinical models).	Multi-institution source slides (from TCGA; 9 institutions noted), public images (TCGA) with curated annotations.	49 pts/50 WSIs, 40× scans; split 36/7/7 (train/val/test).	No (single curated dataset split).	Segmentation (not classification); small test set; TCGA quality variability; no direct subtype labels.

**Table 3:** Future Directions in Deep Learning for Melanoma Subtyping.

Focus Area	Strategies and Technologies	Goals/Expected Outcomes
Multimodal Modeling	Combine histology with genomics (e.g., BRAF, NRAS) Integrate clinical metadata (e.g., age, location, treatment) Use non-invasive imaging (dermoscopy, confocal) Apply GANs and diffusion models for synthetic data Leverage few-shot/zero-shot learning for rare subtypes	Enhance diagnostic accuracy Enable precision dermatopathology Address data imbalance and rare subtype scarcity
Federated Learning and Privacy	Train models across institutions without sharing raw data Use differential privacy techniques	Preserve patient confidentiality Improve generalizability Promote collaboration across centers
Explainable AI (XAI)	Concept bottleneck models Counterfactual explanations Attention-based networks	Increase transparency and interpretability Build pathologist trust Support diagnostic quality control
Clinical Validation and Standards	Conduct prospective clinical trials Evaluate integration into diagnostic workflows Standardize datasets and benchmarks Follow models such as TCGA and ISIC	Demonstrate real-world utility Secure regulatory approval Promote reproducibility and equity
